# Landscape of co-expressed genes between the myocardium and blood in sepsis and ceRNA network construction: a bioinformatic approach

**DOI:** 10.1038/s41598-023-33602-4

**Published:** 2023-04-17

**Authors:** Qi Long, Gang Li, Qiufen Dong, Min Wang, Jin Li, Liulin Wang

**Affiliations:** 1 Department of Critical Care Medicine, Hubei Province Hospital of Traditional Chinese Medicine, 856 Luoyu Street, Wuhan, 430061 Hubei People’s Republic of China; 2 Hubei Province Academy of Traditional Chinese Medicine, 856 Luoyu Street, Wuhan, 430061 Hubei People’s Republic of China

**Keywords:** Biomarkers, Cardiology, Diseases, Risk factors

## Abstract

Septic cardiomyopathy is a serious complication of sepsis. The mechanism of disease pathogenesis, which is caused by infection, is well researched. Despite ongoing efforts, there are no viable biological markers in the peripheral blood for early detection and diagnosis of septic cardiomyopathy. We aimed to uncover potential biomarkers of septic cardiomyopathy by comparing the covaried genes and pathways in the blood and myocardium of sepsis patients. Gene expression profiling of GSE79962, GSE65682, GSE54514, and GSE134364 was retrieved from the GEO database. Student’s *t*-test was used for differential expression analysis. K-means clustering analysis was applied for subgroup identification. Least absolute shrinkage and selection operator (LASSO) and logistic regression were utilized for screening characteristic genes and model construction. Receiver operating characteristic (ROC) curves were generated for estimating the diagnostic efficacy. For ceRNA information prediction, miWalk and lncBase were applied. Cytoscape was used for ceRNA network construction. Inflammation-associated genes were upregulated, while genes related to mitochondria and aerobic metabolism were downregulated in both blood and the myocardium. Three groups with a significantly different mortality were identified by these covaried genes, using clustering analysis. Five characteristic genes—*BCL2A1*, *CD44*, *ADGRG1*, *TGIF1*, and *ING3*—were identified, which enabled the prediction of mortality of sepsis. The pathophysiological changes in the myocardium of patients with sepsis were also reflected in peripheral blood to some extent. The co-occurring pathological processes can affect the prognosis of sepsis. Thus, the genes we identified have the potential to become biomarkers for septic cardiomyopathy.

## Introduction

Sepsis, a multifaceted condition induced by serious infection that results in multi-organ dysfunction and poor clinical outcomes, is responsible for approximately 25 million deaths worldwide^1,2^. Microcirculation disturbance is considered as the most important reason for the refractory shock caused by sepsis. During early sepsis or septic shock, recessive dysfunction of the myocardium is as important and common as the microcirculation disturbance, which is defined as septic cardiomyopathy (SCM). The presence of SCM in septic shock indicates a worse prognosis. SCM raises the death rate of patients with septic shock to 70–90%, and its occurrence ranges from 18–40% in septic shock patients^3,4^. As a result, an early diagnosis of SCM with sufficient accuracy and sensitivity may be beneficial for dealing with the disease. The basic approach for diagnosing SCM is a bedside transthoracic echocardiography, which often shows a left ventricular ejection fraction of 45% with right ventricular dilation^5^. Despite the fact that various laboratory tests, such as atrial brain natriuretic peptide (BNP) and cardiac troponin I (cTnI)^5^, can help in the identification of this disease, the specificity of these biomarkers is still lacking.

Gene sequencing is widely used in clinical and scientific investigation of numerous diseases, including sepsis^6–10^. Compared with peripheral blood, omics studies of the myocardium in sepsis patients are limited. According to a recent study that sequenced the myocardial tissue of sepsis patients, mitochondrial-associated proteins and sarcomere proteins were significantly downregulated in the myocardial tissue of patients with sepsis compared with that of the control group^9^. Although gene sequencing analysis of blood samples from sepsis primarily focused on inflammatory response–related genes and pathways, other investigations have revealed a deficit in leukocyte energy metabolism and variations in mitochondrial levels in sepsis^11,12^. These dysfunctions have been demonstrated to be associated with the incidence of some complications of sepsis, such as acute respiratory distress syndrome (ARDS)^13^. We believe that there are some genes and pathways that change both in the myocardium and peripheral blood in sepsis, in a parallel fashion.

Furthermore, investigations beyond protein-coding RNAs help to show that noncoding RNAs (ncRNAs) are under negative evolutionary selection, implying that they are functional and not transcriptional noise^14^. Since the number and function of protein-coding genomes are strikingly similar between species, ncRNAs have the potential to influence biological processes beyond our understanding^15^. Competing endogenous RNA (ceRNA) networks have been found widely in various disease, including sepsis.

Therefore, our study aimed to compare the gene expression between the myocardium and peripheral blood, tried to identify the genes and pathways that changed in both tissues, and identify potential biomarkers for SCM.

## Results

### DEG identification and GO annotation

**Figure 1:**
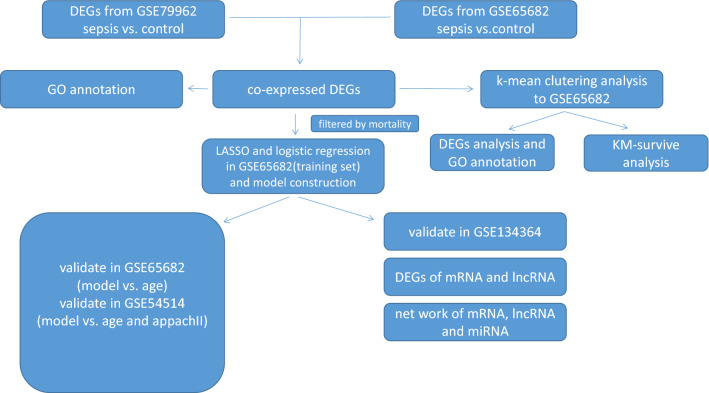
Flow diagram presenting the framework of our study, including the main statistical methods and procedures used.

**Figure 2:**
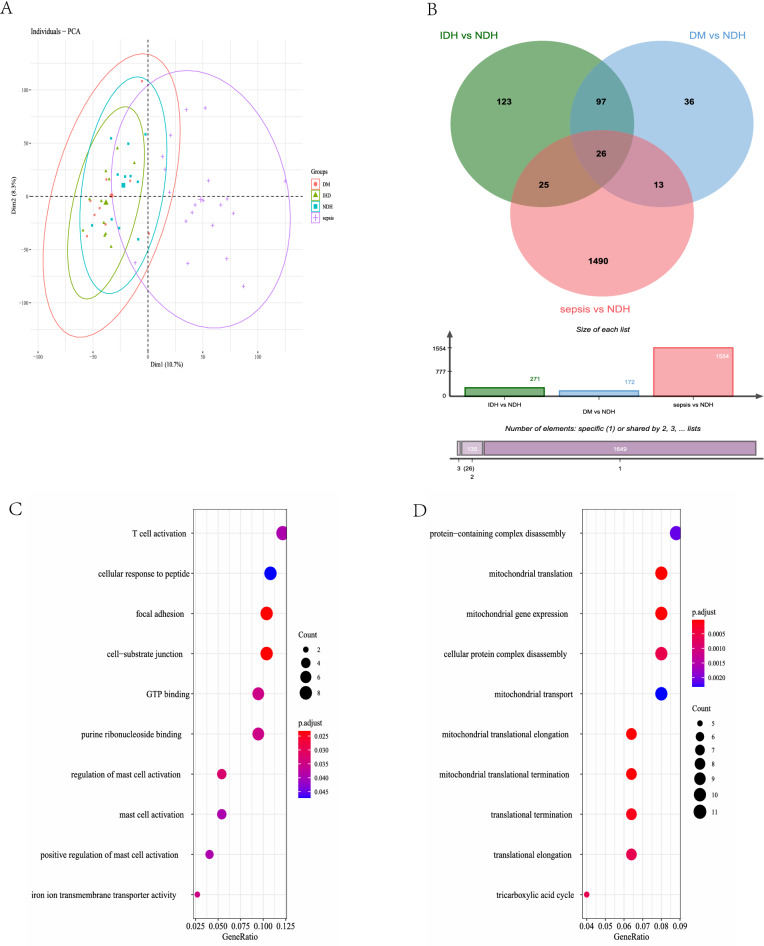
Principal component analysis (PCA) and differentially expressed gene (DEG) identification. (**A**) PCA of the myocardium dataset; the sepsis group is separated from other groups. Dim1 and Dim2 are the principle components 1 and 2 generated by PCA; the distribution plot presents the distribution of different group of myocardium sample. (**B**) Venn diagram of intersection of DEGs between NFH, IHD, sepsis, and DCM. (**C**) Gene ontology (GO) annotation of the common upregulated DEGs. (**D**) GO annotation of the common downregulated DEGs. SCM: septic cardiomyopathy, IHD: ischemic heart disease, DCM: dilated cardiomyopathy, NFH: non-failing heart.

The overall framework of this study is presented as a flow diagram (Fig. 1). In the myocardium dataset (GSE79962), the sepsis group was segregated from other groups as a consequence of principle component analysis (PCA), and no data were discarded (Fig. 2A). We used the non-failing heart (NFH) group as a control and compared subjects with the other three groups independently (Fig. 2B). We acquired 1490 differentially expressed genes (DEGs) that are unique to sepsis, by excluding the DEGs that are common to three disease groups, namely sepsis, ischemic heart disease (IHD), and dilated myocardiopathy (DM). This includes 663 upregulated genes and 827 downregulated genes.

We found 3660 DEGs in the blood dataset (GSE65682), with 1174 upregulated genes and 2486 downregulated genes. Compared with the DEGs from GSE79962 and GSE65682, we obtained 212 genes that were both changed in the same direction in both the myocardium and peripheral blood, in which 132 genes were upregulated and 80 genes were downregulated (Table S1, S2).

We discovered various pathways related to different types of cells and cytokine-related inflammation in upregulated genes in the GO annotation result of the co-expressed DEGs. Pathways related to mitochondrial and protein complex disassembly were enriched in downregulated genes (Fig. 2C,D, Table S3, S4).

### K-means clustering analysis

**Figure 3:**
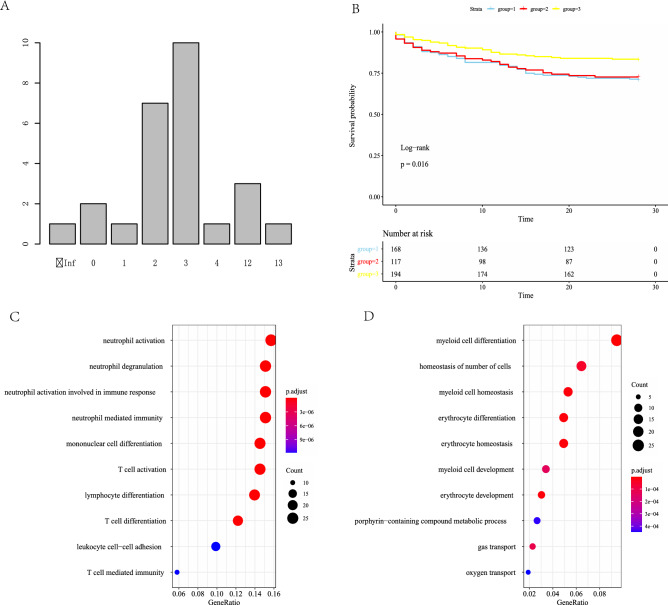
Clustering analysis of sepsis group. (**A**) Barplot from the k-means clustering analysis suggests 3 clusters would be the best classification choice. (**B**) Kaplan–Meier survival analysis showed the mortality of group 3 is significantly higher than that of group 1 and group 2. (**C**) GO annotation of DEGs between group 1 and group 3. (**D**) GO annotation of DEGs between group 2 and group 3.

Using all of the 212 DEGs retrieved, clustering analysis was done on GSE65682. By evaluating the total inside sum of square and average silhouette width, the best number of clusters was determined to be three (Fig. 3A). Class 1 had 168 patients (35.1%), Class 2 had 117 patients (24.4%), and Class 3 had 194 patients (40.1%). Class 1 and Class 2 patients had considerably higher mortality than Class 3 patients (Class 1: 29.2% [49/168], Class 2: 27.3% [32/117], Class 3: 17.0% [33/194]; *p* = 0.016 for chi-squared test). The survival analysis was statistically different (Fig. 3B). The main difference between Class 1 and Class 3 was inflammatory factors, while the difference between Class 2 and Class 3 was more imputed to non-inflammatory variables, such as cell growth, differentiation, homeostasis, and oxygen transport, according to GO annotation of DEGs (Fig. 3C,D, Table S5, S6). We also found differences in DEG expression between Class 1 and Class 2 in terms of inflammatory reaction, cell differentiation, and homeostasis. Because there was no statistical difference in mortality rates caused by these DEGs, we did not further analyze them in this study.

### Development of class model

**Figure 4:**
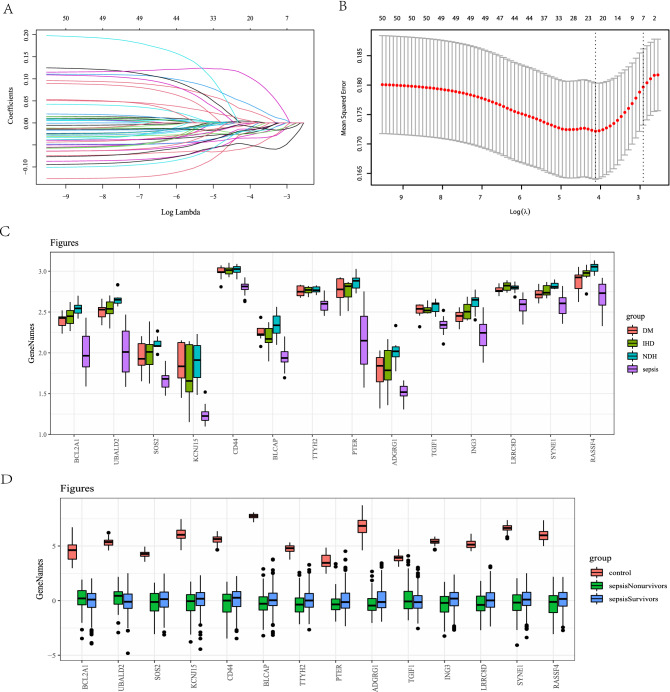
(**A**) Lasso regression of the variable selection. (**B**) The area of best λ values varies from 0.009211057 to 0.05394935, the best variable numbers should be in the area from 7–21. (**C**) Boxplot of hubgene expression in the myocardium with the data in GSE79962. (**D**) Boxplot of hubgene expression in the myocardium with the data in GSE65682.

Using univariate logistic regression, 212 co-expressed DEGs were filtered by mortality. Fifty genes were found to be linked to mortality and were analyzed using LASSO, and the best tuning parameter was suggested to be 0.009211057–0.05394935. In order to match the sample size in the training dataset, we adjusted the area for variable selection and 14 variables were acquired using the LASSO regression model (Fig. 4A,B, Table S7). Finally, the 14 most essential LASSO classifier variables were maintained and used to create logistic regression models. The expression of the 14 hubgenes in both myocardium and blood samples are presented in Fig. 4C and Fig. 4D. In the regression model, five genes were maintained (*BCL2A1*, *CD44*, *ADGRG1*, *TGIF1*, and *ING3*) after the multivariable logistic regression analysis.

### Model validation

**Figure 5:**
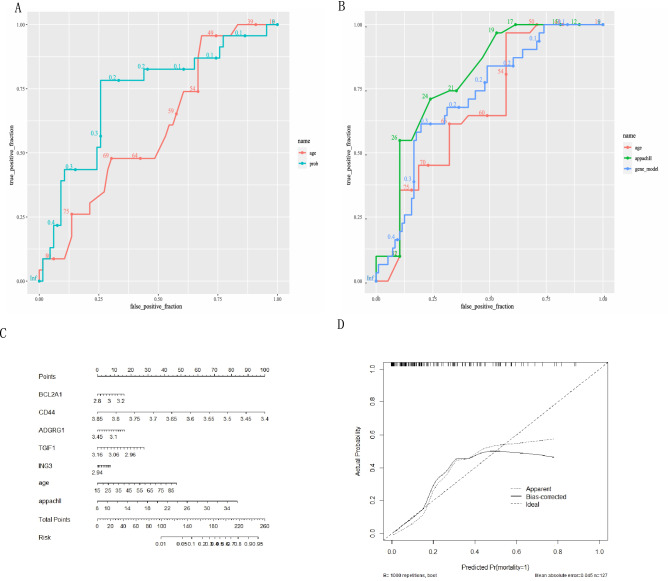
(**A**) Mortality prediction of gene model and age in testing dataset from GSE65682, AUC for age was 0.585 (95% CI 0.247–0.911) and the AUC for the five-gene-age model was 0.669 (95% CI 0.782–0.565). (**B**) Mortality prediction of gene model, age and Apache II scoring system in testing dataset from GSE54514. The AUC of the five-gene model was 0.718 (95% CI 0.802–0.613), the AUC of age was 0.676 (95% CI 0.427–0.968), and the AUC of APACHE II was 0.792 (95% CI 0.760–0.710). (**C**) Nomogram generated from GSE54514 integrating the risk factors available, including APACHE II scoring system, age, and five-gene model. (**D**) Calibration curve testing of nomogram generated from GSE54514.

We tested the predictive potential of age on mortality in the produced dataset, finding that the area under the curve (AUC) for age was 0.585 (95% CI 0.247–0.911) and the AUC for the five-gene-age model was 0.669 (95% CI 0.782, 0.565). (Fig. 5A). The six-gene model outperformed age (*p* < 0.001).

In the external validation set GSE54514, the same procedure was used. The GSE65682 five-gene model did not match well in GSE54514 (AUC = 0.478; 95% CI 0.969, 0.129). We retrained the five genes to mortality in GSE54514, considering the altered data background, and the results improved dramatically. In GSE54514, we compare the five-gene model with the age and APACHE II scoring system in terms of mortality prediction efficiency. The AUC of the five-gene model was 0.718 (95% CI 0.802–0.613), the AUC of age was 0.676 (95% CI 0.427–0.968), and the AUC of APACHE II was 0.792. (95% CI 0.760–0.710). The five-gene model's death prediction effectiveness was better than age but slightly lower than APACHE II (Fig. 5B). Figure 5C and D show the nomograph and calibration curves, which were used to integrate genes with age and the APACHE II scoring system to predict mortality.

### ceRNA network construction

**Figure 6:**
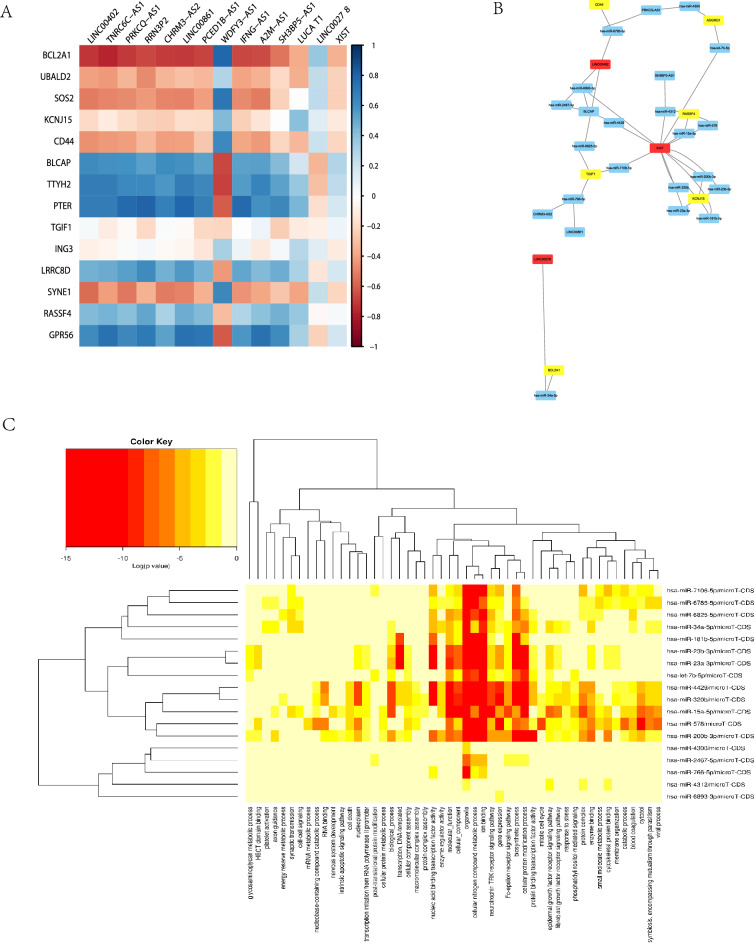
ceRNA network construction. (**A**) Corrplot of mRNA and lncRNA in GSE134364. (**B**) Network generated by Cytoscape of ceRNA. (**C**) Functional enrichment analysis of miRNAs identified using the GO database.

In the GSE1343641 dataset, 1,418 long noncoding RNAs (lncRNAs) were found. Using the adjusted *p* value < 0.001, we compared the differential expression of these lncRNAs in sepsis and the control group with the limma program and 14 differentially expressed lncRNAs were identified (Table S8). The correlation between the expression levels of mRNA and lncRNA is shown in Fig. 6A. We used miWalk to retrieve the information on miRNA and microRNA (miRNA). We chose TargetScan and MirtarBase, two important miRNA databases, to perform the prediction in miWalk. We also used lncBase to get the lncRNA-miRNA information and obtained miRNAs related to both lncRNA and mRNA. The network among lncRNA, mRNA, and miRNA is displayed in Fig. 6B. LINC00278, XIST, and LINC00402 are lncRNAs that are closely associated with the identified miRNAs, i.e., CD44, ADGRG1, RASSF4, TGIF1, KCNJ15, and BCL2A1. Functional enrichment analysis was performed on 18 miRNAs using GO. In addition to inflammation and energy metabolism, the functions related to these miRNAs involve many other aspects that reflect the complexity of miRNA functions, such as signal transduction and cellular components. The result of the functional enrichment analysis is presented in Fig. 6C.

## Materials and methods

### Patients and datasets

Our study used four datasets (GSE79962, GSE65682, GSE54514, and GSE134364), with all RNA sequencing data and clinical information coming from the Gene Expression Omnibus (GEO) database.

GSE79962 contains 51 myocardium samples, comprising 20 samples of SCM, 11 samples of IHD, 11 samples of dilated cardiomyopathy (DCM), and 9 samples of NFH.

We collect 802 whole blood samples for sepsis patients (n = 760) and healthy controls (n = 142) in the blood dataset GSE65682. In total, 468 sepsis samples with 28-days survival data were studied as training dataset and internal testing dataset for model construction with sepsis survivors (78.0%, 365/468), and sepsis non-survivors (22%, 103/468).

GSE54514 included 163 daily PAX gene samples from sepsis survivors (n = 26), non-survivors (n = 9), and healthy controls (n = 18) for up to 5 days and was used as external testing dataset.

GSE134364 contains 298 samples analyzed by protein-coding and ncRNA expression (GPL17586). This dataset was used for ceRNA network construction.

### DEG analysis and functional enrichment analysis

We used the limma package in R to perform the differential expressed analysis^16^. All *p*-values were corrected using the Benjamini–Hochberg approach.

In the myocardium dataset (GSE79962), we used the adjusted *p*-value < 0.01 criteria alone rather than compared with the fold change value to keep additional genes for future analysis. To examine the distribution of the four sets of samples in myocardium data, we first used PCA. We compared the non-falling heart group's gene expression to that of the other three groups (sepsis, IHD, and DCM), and maintained the dysregulated genes that were unique to SCM and not seen in IHD or DCM patients using a Venn diagram^17^.

We then compared the sepsis group to the healthy control group in GSE 65,682 and discovered the sepsis DEGs (adjusted *p*-value < 0.01 and |logFC|> 0.5).

DEGs from the myocardium and peripheral blood were compared, and genes that were commonly up- or downregulated were recorded. Despite the probability of retroregulation, we continue to focus on changes in the same direction in both the heart and the blood to avoid interference. Using the "clusterProfile" package in R, we ran Gene Ontology (GO) annotation on the co-expressed DEGs^18^.

### K-means clustering analysis

In 468 septic patients from GSE65682, the co-expressed DEGs were employed for k-means clustering analysis. The elbow and average silhouette method were used to determine the number of clusters. DEGs from distinct groups were compared (adjusted *p*-value < 0.01, |logFC|> 1) and GO annotation was received. The clustering was done using the R package NbClust^19^.

### Variables selection and model construction

In the GSE65682 deviation data set, all co-expressed DEGs were filtered to univariate logistic regression on mortality with an adjusted *p*-value < 0.05. The least absolute shrinkage and selection operator (LASSO) was then utilized to find relevant classifier variables because there were still more than 50 variables left. Glmnet packages in R were used to perform LASSO regression analysis^20^. The expression of hubgenes selected by LASSO is visualized by a boxplot in both the myocardium and blood datasets.

In a 7:3 ratio, GSE65682 was divided into a training set and a testing set. Backward stepwise regression to mortality was performed using the top genes derived from LASSO. Nested logistic regression models were created by sequentially deleting variables until all variables in the model were statistically significant with a *p*-value < 0.01. The prediction efficiency of several models was compared using the chi-squared test.

### Model validation

Age and APACHE II score were risk factors available in our databases that were confirmed to be related to sepsis mortality. Furthermore, we compared the mortality prediction efficiency between the model and these risk factors. Using the AUC of the ROC curve, the gene model was first verified to predict the mortality-forecasting efficiency of the test dataset generated in GSE65682 and then to predict mortality with age. We utilized the same strategy in GSE54514 to assess the gene model's accuracy in predicting mortality with age and APACHE II score. We also used nomograph and calibration curves to evaluate the weight of several factors in predicting death by combining the genes with age and APACHE II score. The pROC package in R was utilized for the analysis^21^.

### ceRNA network construction

DEGs of lncRNA were obtained in GSE134364 using differential expression analysis. We set |logFC|> 2 and adjusted *p*-value < 0.01 to get the correct number of DEGs with high expression levels of lncRNA. Correlation analysis between the expression levels of lncRNAs and mRNA were utilized using Pearson’s correlation coefficients and visualized by heatmaps. We applied package pHeatmap in R to generate the heatmap here (Fig. 6A).

To build the regulatory network, we predicted the possible relation in mRNA-miRNA and lncRNA-miRNA by using some miRNA and ncRNA related databases and web tool. We first obtained the information of mRNA-miRNA interaction in miWalk, a useful web tool to get miRNA relation information^22^. In miWalk, you can use information from databases to predict the interaction between miRNA and other important molecules. Hence, we chose miTarbase and TargetScan to predict mRNA-miRNA interaction. We set 5-UTR as the cristae to select our target miRNA. We also obtained the lncRNA-miRNA interact information in lncBase. We used data medium intensity correlation to obtain our target miRNA that might be related with our lncRNA from GSE134364^23^.

The miRNA related to both lncRNA and mRNA were applied to preform functional enrichment. We used mirPath to finish the functional enrichment analysis and generate the heatmap in Fig. 6C^24^. Network analysis was finished by R and Cytoscape.

## Discussion

In our study, we analyzed the DEGs common in the myocardium and peripheral blood and saw that upregulated genes were associated with inflammatory cell activation and expression of inflammatory factors, while downregulated genes were associated with mitochondrial function and aerobic metabolism. We performed unsupervised classification learning of patients with sepsis using these common DEGs, and the results were obtained for three different groups of people. We observed no significant difference in mortality between groups 1 and 2, while mortality increased nearly twice in these two groups compared to group 3. We compared the gene expression of group 1, group 2, and group 3, obtained their DEGs, and using GO annotation, we could see that the differential genes in group 1 and group 3 were mainly in the activation of inflammatory cells, while the differential genes in group 2 and group 3 were mainly focused on cell differentiation, homeostasis, and aerobic respiration. Our results support the conclusion that the inflammatory storm is a major factor in sepsis's poor prognosis^25^. Bioinformatic analyses have attempted to identify sepsis patients based on their inflammatory levels, and have discovered that individuals with greater inflammatory levels have a poorer prognosis than those with a normal inflammatory response^11,26,27^. Our study also suggests that changes in aerobic metabolism, mitochondrial function, and cell membrane stability, in addition to the intensity of inflammatory response, may affect sepsis mortality, a process that may also be involved in the development of SCM.

Energy metabolism proteins such as mitochondria related protein and cardiac contractility related proteins have been proven to be considerably downregulated in the myocardium tissue of sepsis patients^9^. In our research, we demonstrated that mitochondrial dysfunction and metabolism abnormalities co-exist in cardiac muscle and blood cells, which may have been overlooked in the onset and progression of SCM. In actually, mitochondrial damage and energy metabolism problems are systemic disorders in sepsis. Other organs and tissues recovered from septic patients, such as skeletal muscle^28–30^, platelets^31–33^, leukocytes^11^, and peripheral blood mononuclear cells (PBMCs) ^34,35^, have shown mitochondrial dysfunction. Plasma mitochondrial DNA levels have even been linked to the development of ARDS in trauma and sepsis patients^13^.

We applied these hubgenes for machine learning of mortality outcomes in our modeling method, and the final model predicted death with somewhat better accuracy than age and poorer than the APACHE II score, with acceptable predictive efficiency but space for improvement.

Because there is not much variation in coding RNAs across species, it is currently thought that ncRNAs (lncRNAs and sncRNAs) may play a critical regulatory role in many diseases^14^. Although there are many studies on ncRNAs, the research on the function and functional annotation of ncRNAs is still in its infancy^36,37^. One study of ncRNAs in sepsis showed that changes in ncRNAs were not consistent with changes in coding RNAs, but had different individual differences^38^. This conclusion was supported by our research results. In our study, the expression levels of mRNA and lncRNA were not completely correlated. Furthermore, it can be seen that among the five coding RNAs that constitute the model, the correlation between *TGIF1*, *ING3*, and the most differentially expressed lncRNAs was not significant. At the same time, LINC00278 and XIST were also weakly correlated with mRNA. Additionally, the functional enrichment analysis of identified miRNA was very different from that of mRNA. Inflammatory-related mRNAs were identified as independent risk factors for sepsis (group 1 vs. group 3). In the non-inflammatory mRNA groups (group 2 vs. group 3), cell differentiation, development, and homeostasis were the main functions changed, and some aerobic metabolism-related functions were included. However, the same functions changes have not been obviously observed in miRNA enrichment analysis. In other words, the results of the functional analysis of miRNA lacked specificity compared with those of mRNA.

In addition to the lack of research on ncRNA, especially on sepsis, the lack of specificity of the miRNA enrichment analysis may be caused by the complexity of miRNA regulatory networks. Pools of RNA molecules, however, can act as competing endogenous RNAs (ceRNAs) and influence their expression levels indirectly by competitively binding shared miRNAs. MiRNAs can act as decoys by binding multiple RNAs, and RNAs can act as ceRNAs by competing for binding multiple miRNAs, resulting in numerous cross-talk interactions that may favor significant large-scale effects despite the weakness of single interactions^39^. Therefore, we predicted the possible relation between mRNA-miRNA and lncRNA-miRNA to further elaborate the network relationships between ceRNAs. This research approach has been used in many fields to validate the disease development process and the gene modules associated with the disease^40–42^. Therefore, we applied this research approach in our study and also tried to understand the network associated with ceRNA in septic cardiomyopathy in a holistic manner.

There are a few limitations in our research. Firstly, many publicly accessible sepsis statistics focused exclusively on the differential diagnosis of sepsis versus non-infectious systemic inflammatory response syndrome and did not include death data. Since it was difficult to obtain a database with adequate findings from cardiac ultrasonography and other laboratory tests, comparing our model to these standard procedures in terms of diagnostic efficiency and mortality prediction was problematic.

There is still more work to be done in the field of omics in SCM. More laboratory and clinical evidence on myocardial damage is needed, as well as a better prospective research design that includes a precise description of SCM. Similarly, we hope to obtain blood and heart tissue samples from the same patient to complete the design of a matched experiment; however, this is quite challenging.

The method we used for the omic study is similar to that of a retrospective study. Therefore, the resulting hubgene does not have a strong evidence-based grade. In terms of specific mechanisms, the functions of these biomarkers need to be further explored in animal and in vitro experiments. Furthermore, regarding ultimate clinical effects, more prospective randomized controlled trials are needed for validation. In conclusion, our study only screened a portion of hubgene with biomarker potential from previous sequencing data, and there is still much work to do before practical clinical application. However, we hope that our study will provide useful information for subsequent studies to identify potential biological markers for septic cardiomyopathy.

## Conclusion

In myocardial and blood tissues of patients with sepsis, some pathways and genes regarding inflammatory cell activation are co-regulated and some non-inflammatory–related cellular pathways and genes are downregulated, such as cellular homeostasis, mitochondrial function, and aerobic respiration. These genes are associated with mortality in patients with sepsis. Impaired mitochondrial function and aerobic respiration co-exist in the heart muscle and blood in sepsis. There is a link between those coding RNAs and ncRNAs that are most significantly altered in sepsis patients, but some genes exist independently of each other.

## Supplementary Information

Supplementary Information.

## Data Availability

Our study used four datasets (GSE79962, GSE65682, GSE54514, and GSE134364), with all RNA sequencing data and clinical information coming from the Gene Expression Omnibus (GEO) database.
